# Notch signaling governs the expression of glypican Dally to define the stem cell niche

**DOI:** 10.1242/bio.047696

**Published:** 2020-01-10

**Authors:** Songhua Zhao, Chan Wu, Zhiyang Gao, Xin Li, Zheng Guo, Zhaohui Wang

**Affiliations:** 1 State Key Laboratory of Molecular Developmental Biology, Institute of Genetics and Developmental Biology, Chinese Academy of Sciences, Beijing 100049, China; 2 The University of Chinese Academy of Sciences, Beijing 100049, China

**Keywords:** Stem cell niche, Notch signaling, Glypican, Human GPC

## Abstract

Extracellular glypicans play pivotal roles in organogenesis, stem cell maintenance and cancer development. However, the growth phenotypes associated with different levels of glypican are not consistent in development or tumorigenesis. This requires clarification on how the spatial patterns of glypican relate to the distribution of signaling molecules in different cellular contexts, and how glypican expression is regulated. We have previously reported that Dally, one of the glypican members in *Drosophila*, is required in the niche for the maintenance of germline stem cells (GSCs) via short-range BMP signaling in ovary. However, the regulatory mechanism of glypican pattern in the ovarian stem cell niche remains elusive. Our current data demonstrate that the Notch pathway is genetically upstream of Dally and its function to maintain GSCs relies on Dally expression. Combining yeast and fruit fly genetics, we illustrate that Dally is under the transcriptional control of Notch signaling via the transcription factor Su(H). Further, we assayed human glypicans and disease-associated variants in *Drosophila* ovary, which can serve as an effective system to evaluate the structure–function relationship of human homologs.

## Abstract (precis)

**Summary:** Spatial regulation of a cell surface glycoprotein defines the territory of germline stem cells.

## INTRODUCTION

The extracellular matrix exists in virtually all multicellular organisms, and organizes an environment that influences the survival, division, differentiation, migration and many other functions of the cells in contact. Proteoglycans and fibrous proteins are the two classes of macromolecules in the matrix. The core protein of proteoglycan is covalently modified by long unbranched polydisaccharide chains called glycosaminoglycans. Glypican is a subtype of heperan sulfate proteoglycans anchored on cell surface through the glycosylphosphatidylinositol (GPI) attached to the carboxyl-terminus of the core protein (Li et al., 2011; Filmus and Capurro, 2014).

Due to their structural features and extracellular localization, glypicans are involved in many cell-signaling pathways, including Wnt, Hh, FGF, cytokine and BMP, during development. Mutations in human glypican genes are closely associated with growth anomalies such as Simpson-Golabi-Behmel Syndrome (SGBS, overgrowth) or omodysplasia (bone undergrowth) (Pilia et al., 1996; Campos-Xavier et al., 2009). Consistently, mouse models of glypican mutants demonstrate similar abnormalities caused by the disruption in Wnt or Hh signaling (De Cat et al., 2003; Capurro et al., 2017). In *Drosophila*, where glypicans have been intensively studied, glypican Dally or Dally-like is necessary for the diffusion, stability and/or reception of the extracellular signaling molecules (Yan and Lin, 2007; Wu et al., 2010; Yan et al., 2010; Pennetier et al., 2012; Nakato and Li, 2016). Dally can physically bind Dpp (fly BMP) or Upd (fly cytokine), the distribution of which in a tissue can be modified by Dally alterations (Belenkaya et al., 2004; Akiyama et al., 2008; Hayashi et al., 2012; Zhang et al., 2013). The distinct expressions of Dally in wing and haltere modulate Dpp diffusion to realize the divergent morphology of these appendages (Crickmore and Mann, 2007).

The spatial and temporal patterns of glypican are critical to regulate multiple signaling pathways, and its expression is predominant in early development (Iglesias et al., 2008; Filmus and Capurro, 2014; Fujihara and Ikawa, 2016; Li et al., 2018). It is not unexpected that aberrant glypican expressions were observed in tumorigenesis (Li et al., 2018; Theocharis and Karamanos, 2019). For example, human glypican 3 (GPC3) is upregulated in most hepatocarcinoma and indicative of differentiation grade, but is downregulated in some non-liver tumors including ovarian or breast tumors (Kaseb et al., 2016). In GPC3-transgenic mice, increasing GPC3 actually inhibited hepatocyte proliferation and liver regeneration (Liu et al., 2010a). Apparently, the growth phenotypes associated with different levels of glypican are not consistent in development or tumorigenesis. This requires clarification on how spatial patterns of glypican relate to the distribution of signaling molecules in different cellular contexts, and how glypican expression is regulated.

We have previously identified Dally as a key factor defining the range of germline stem cells (GSC) in *Drosophila* ovary (Guo and Wang, 2009; Hayashi et al., 2009; Liu et al., 2010b). Dally expression is normally restricted to a few somatic cells adjacent to GSCs, and manipulating the spatial pattern of Dally is sufficient to change the size of the GSC niche. Here, we take advantage of Dally's pattern–function relationship in the *Drosophila* GSC niche, and found that Notch signaling is required for Dally expression, while Dally is the mediator for the Notch pathway to achieve its function in maintaining ovarian GSCs. Additionally, we have evaluated the structure–function relationship of human glypicans and the disease-associated mutant forms in the developmental context of GSC niche.

## RESULTS

### *Notch*-specified GSC niche relies on the function of *dally*

In *Drosophila* ovaries, Notch signaling specifies the cap cells (Song et al., 2007), the major components of the GSC niche, and glypican Dally is specifically expressed in the cap cells and is required to define the GSC range (Guo and Wang, 2009; Hayashi et al., 2009). We speculate that Dally expression may be under the control of Notch signaling. First, we examined the genetic relationship between *Notch* and *dally*.

**Fig. 1.**
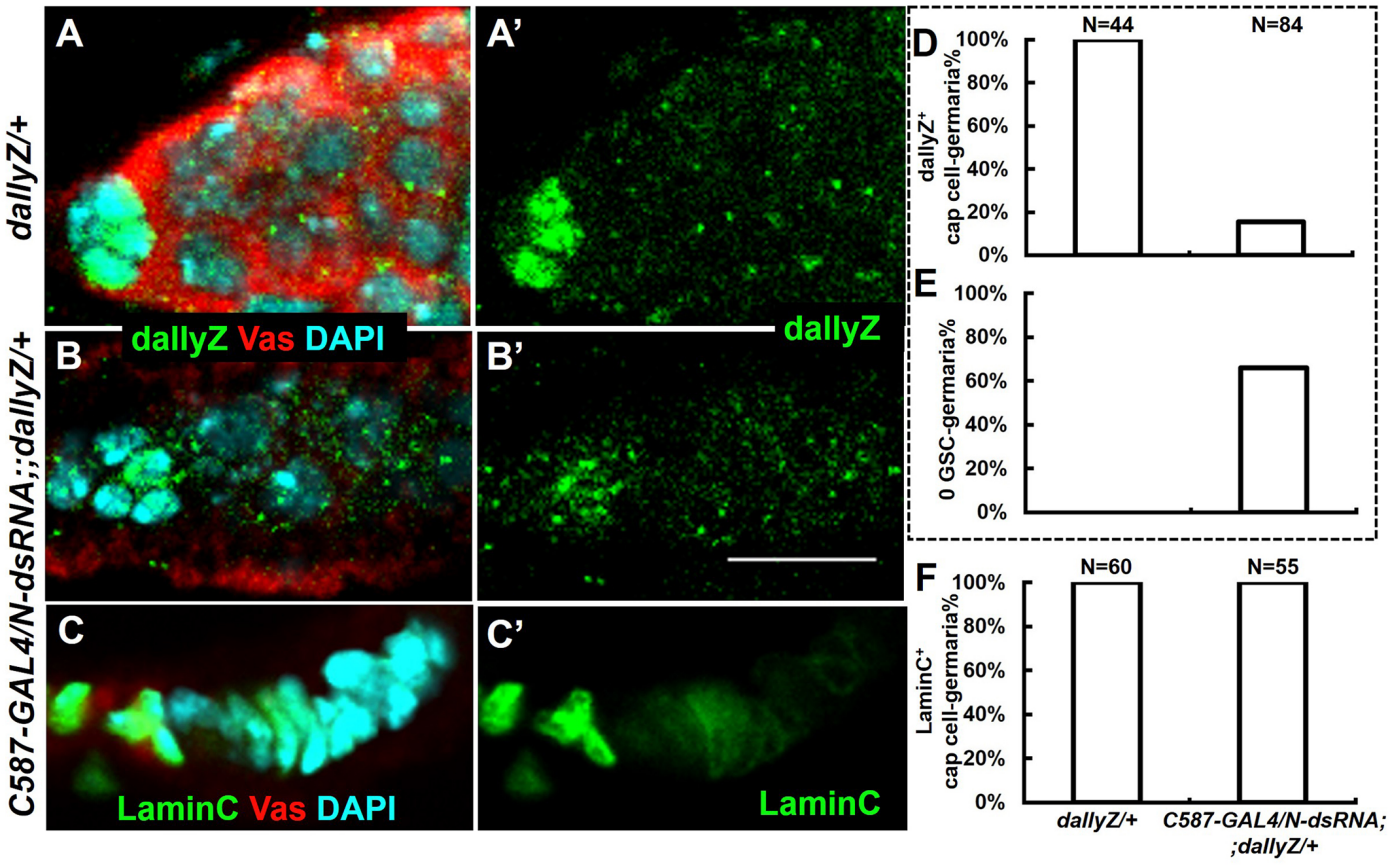
**Knockdown of *Notch* reduced** ***dally* transcription in the ovarian GSC niche.** (A) LacZ-reflecting *dally* transcription is expressed in the cap cells of the GSC niche in the *dallyZ/+* ovary. Only the anterior tip of an ovariole, also known as the germarium, is shown. (B) Knockdown of *Notch* (*C587-GAL4/N-dsRNA;;dallyZ/+*)-induced dallyZ loss in the cap cells. (C) LaminC labels cap cells of the niche (*C587-GAL4/N-dsRNA;;dallyZ/+*), while no germ cells were left in this germarium. (D–F) Genotypes are shown in panel F. N, number of gemaria scored. Same samples were scored for different features in D and E. (D) Scores of germaria containing dallyZ shown in panel A′ and B′. A germarium of ‘dallyZ^+^’ was determined by the presence of at least one cap cell containing dallyZ signal in a germarium. (E) Scoring 0 GSC-germaria of *dallyZ/+* or *C587-GAL4/N-dsRNA;;dallyZ/+* ovaries. Empty germarium without Vas-positive single cells was counted as ‘0 GSC’. (F) Scores of germaria containing LaminC shown in panel C. A germarium of ‘laminC^+^’ was determined by the presence of at least one cap cell containing LaminC signal in a germarium. Note: crosses were raised at 25°C for 7 days, then transferred to 30°C until eclosion, and the adult flies were maintained at 30°C for another 10 days before dissection. Vas, a germline-specific marker. Scale bar: 10 μm.

**Fig. 2.**
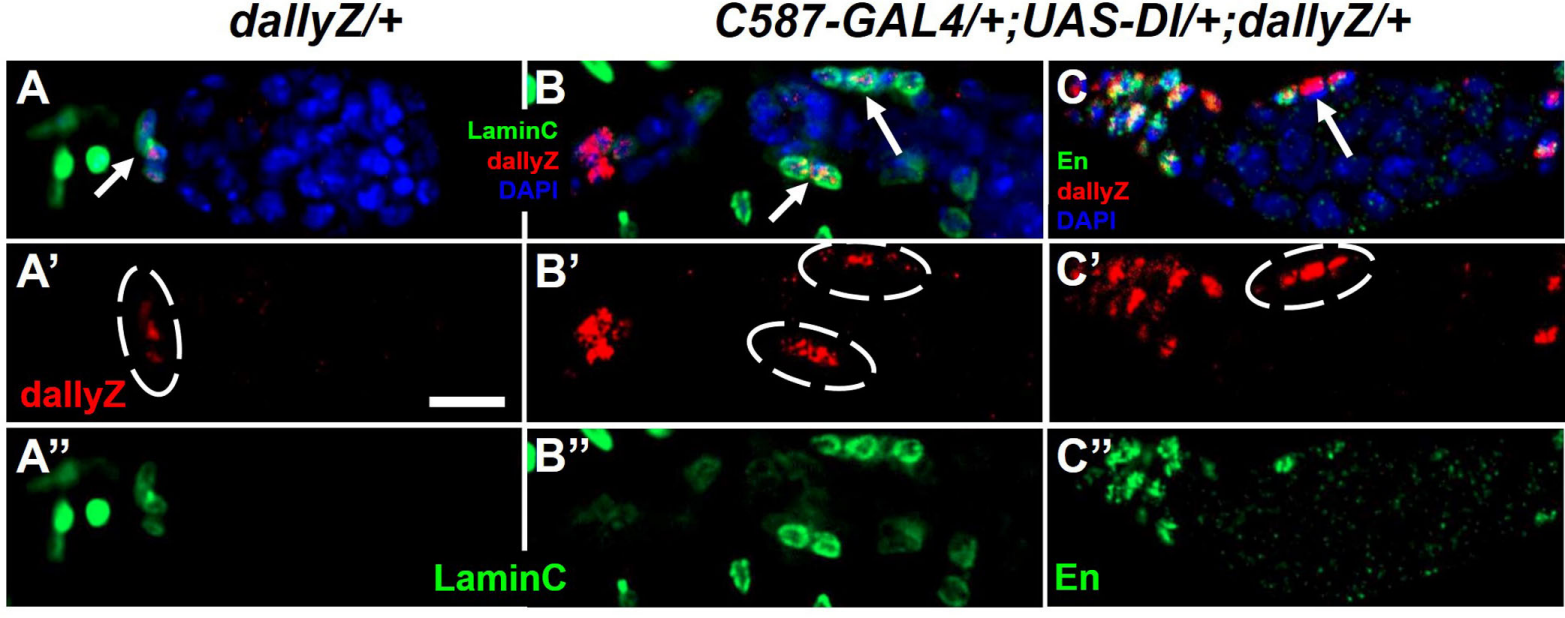
**Dally was expressed in ectopic cap cells induced by Notch's ligand*.*** (A) In a wild-type germarium, dallyZ is present in the cap cells, which are marked by laminC (circled). (B,C) Ectopic activation of the Notch pathway by ligand Dl in the somatic cells of germarium led to dallyZ expression in those extra cap cells. Arrows or circles indicate dallyZ-positive cap cells. Scale bar: 10 μm.

Is Notch signaling required for Dally expression? We reduced Notch level in somatic cells by RNAi and checked the transcription reporter of *dally* (Fig. 1, *dallyZ*). Normally *dallyZ* staining is present in the cap cells (Fig. 1A), but markedly diminished upon Notch-RNAi (Fig. 1B,D). Consistently, the function of the GSC niche was also compromised, as revealed by the rate of GSC loss (Fig. 1E). This lack of Dally expression was not due to the absence of cap cells, which could still be identified by the cap cell marker LaminC (Fig. 1C,F). However, *dallyZ* was activated in ectopic cap cells induced by expressing Dl, a Notch ligand, in an expanded region beyond the GSC niche (Fig. 2). Thus, Notch signaling is both necessary and sufficient for Dally expression in the GSC niche, whereas the changes of Notch pathway components were not detected in *dally* mutants (Fig. S1).

**Fig. 3.**
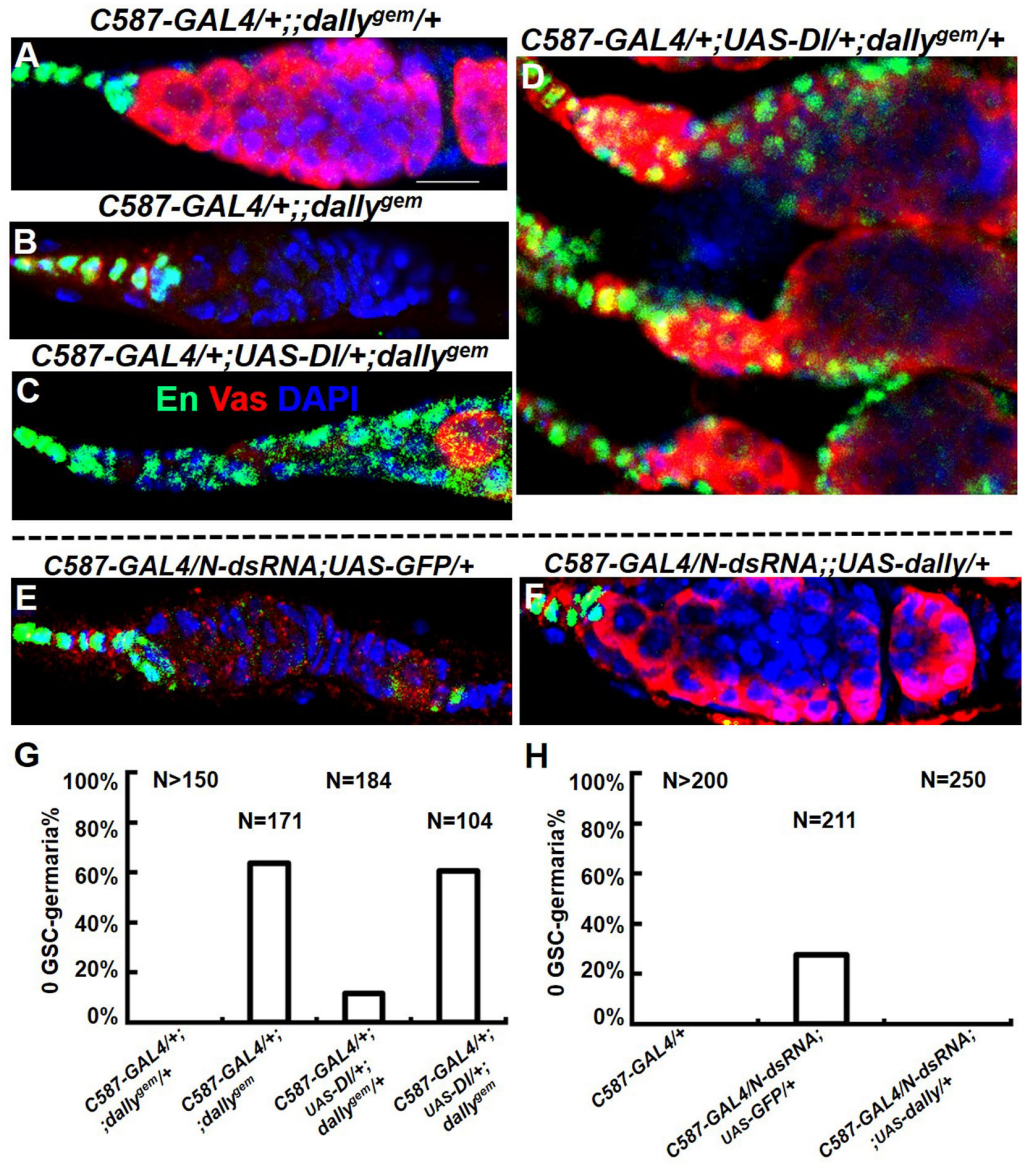
***dally* is genetically downstream of the Notch pathway in defining GSC niche.** Color code indicated in panel C is valid for A–F. En (Engrailed), a marker for cap cells; Vas, germline marker. (A) The normal germarium is filled with Vas-positive germ cells. (B) No germ cell was present in *dally^gem^* germarium. (C) Germ cell loss could not be rescued by extra cap cells induced by Dl in *dally* mutant background. (D) Extra germ cells were induced by extra cap cells (expanded En+ cells). (E) Most germ cells (including GSCs) were absent in this germarium upon somatic RNAi of Notch (N-dsRNA). (F) Germline loss shown in E was rescued by somatically expressed Dally. (G,H) N, number of gemaria scored. Empty germarium without Vas-positive single cells was counted as ‘0 GSC’. (G) Percentage of 0 GSC-germaria shown in panels A–D. (H) Percentage of 0 GSC-germaria shown in panels E–F. Crosses for panels E and F were raised at 25°C for 7 days, then transferred to 30°C until eclosion, and the adult flies were maintained at 30°C for another 10 days before dissection. Scale bar: 10 μm.

To clearly demonstrate if *dally* is downstream of *Notch* in the ovarian GSC niche, we tested if Dally expression is sufficient to rescue GSC loss caused by compromised Notch signaling. Consistent with previous reports, disrupting either Notch or Dally expression resulted in GSC loss (Fig. 3B,E), and the latter case could not be reversed by overexpressing the Notch ligand Delta (Dl) though ectopic cap cells were induced (Fig. 3C, En-positive cells). Overexpressing Delta did induce both ectopic cap cells and GSCs when Dally is present (Fig. 3D). Most importantly, GSCs were significantly restored by Dally expression in the background of Notch-RNAi (Fig. 3F,H). Thus, we demonstrated that *dally* is genetically downstream of Notch signaling in the ovarian GSC niche.

### Transcription of Dally is under the control of the Notch pathway in the ovarian GSC niche

**Fig. 4.**
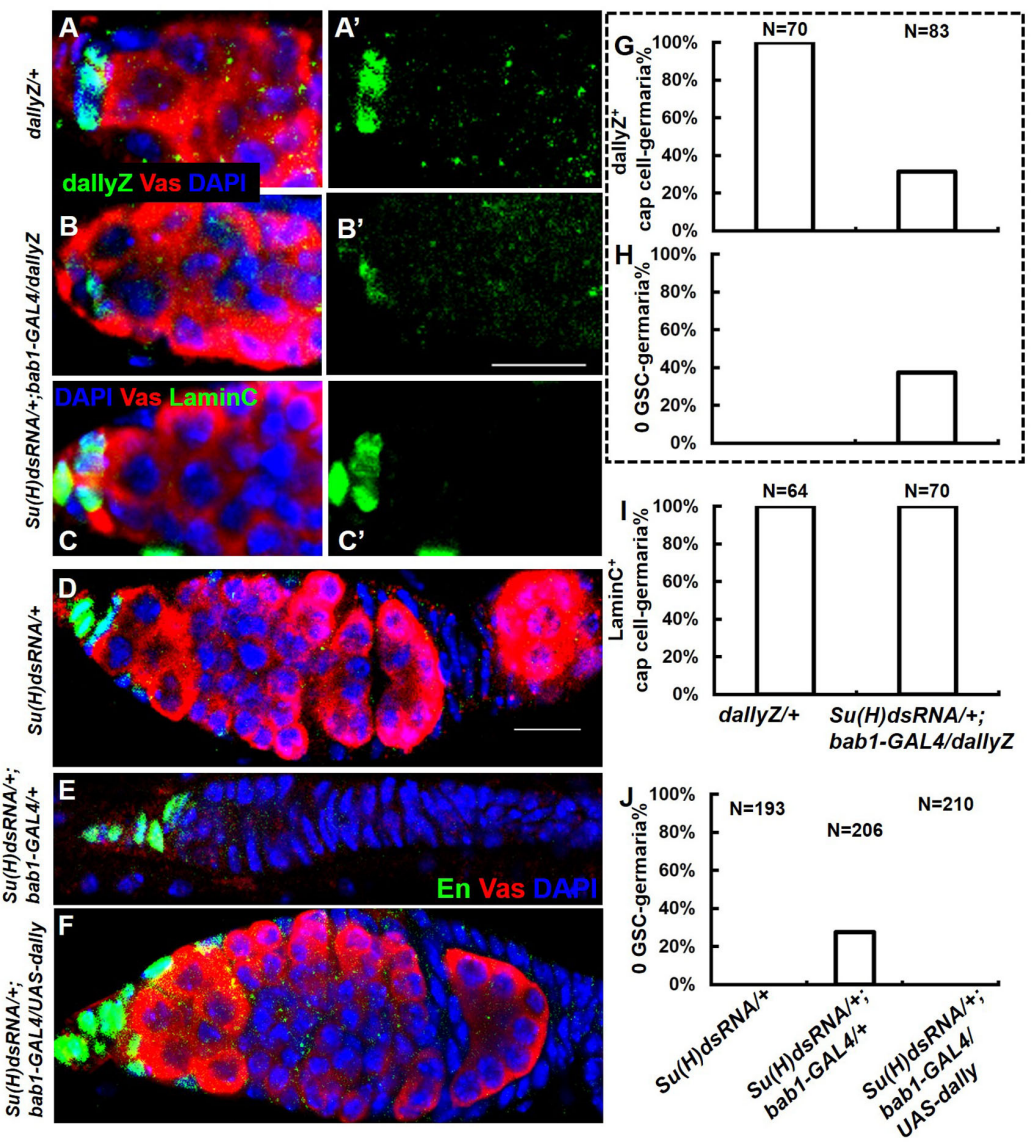
***Su(H)* is required for the expression and function of *dally* in the GSC niche.** (A) Dally reporter is expressed in the cap cells of the GSC niche in the control (*Su(H)dsRNA/+; +/dallyZ*) ovary. (B) Knockdown of Su(H) (*Su(H)dsRNA/+; bab1-GAL4/dallyZ*) led to dallyZ loss in the cap cells, which were separately labeled by LaminC shown in panel C. (D–F) Color code for immunostained signals is shown in panel E. (D) The control germarium is filled with Vas-positive germ cells. (E) No germ cell was present in the germarium, when Su(H) was reduced by *bab1-GAL4*-driven RNAi in the terminal filament and cap cells. (F) Germline loss caused by Su(H) RNAi could be rescued by Dally expression in the same cells. (G–I) Genotypes are shown below the X-axis of panel I. N, number of germaria scored. Same samples were scored in G and H. Empty germarium without Vas-positive single cells was counted as ‘0 GSC’. (G) Score of germaria containing dallyZ shown in panel A′ and B′. (I) Score of germaria containing LaminC shown in panel C′. (J) Scoring 0 GSC-germaria of samples shown in panel D–F. Crosses were raised at 25°C for 4 days, then transferred to 30°C until eclosion, and the adult flies were maintained at 30°C for another 5 days before dissection. Scale bars: 10 μm.

Su(H) is the well-characterized transcription factor downstream of Notch. To examine its relationship with the expression and function of Dally in the GSC niche, we used a niche-specific driver [bab1-GAL4, active in cap cells and terminal filaments (Bolívar et al., 2006; Guo and Wang, 2009)] to knock down Su(H). As expected, not only the signal of Dally reporter was evidently reduced in the cap cells (Fig. 4B,G), but GSCs were also lost in about 40% of the germaria (Fig. 4E,H,J). The lack of signal in the GSC niche was not due to complete loss of cap cells, which were present in all germaria examined (Fig. 4C,I). Resupplying Dally reversed GSC loss caused by Su(H) RNAi (Fig. 4F,J).

**Fig. 5.**
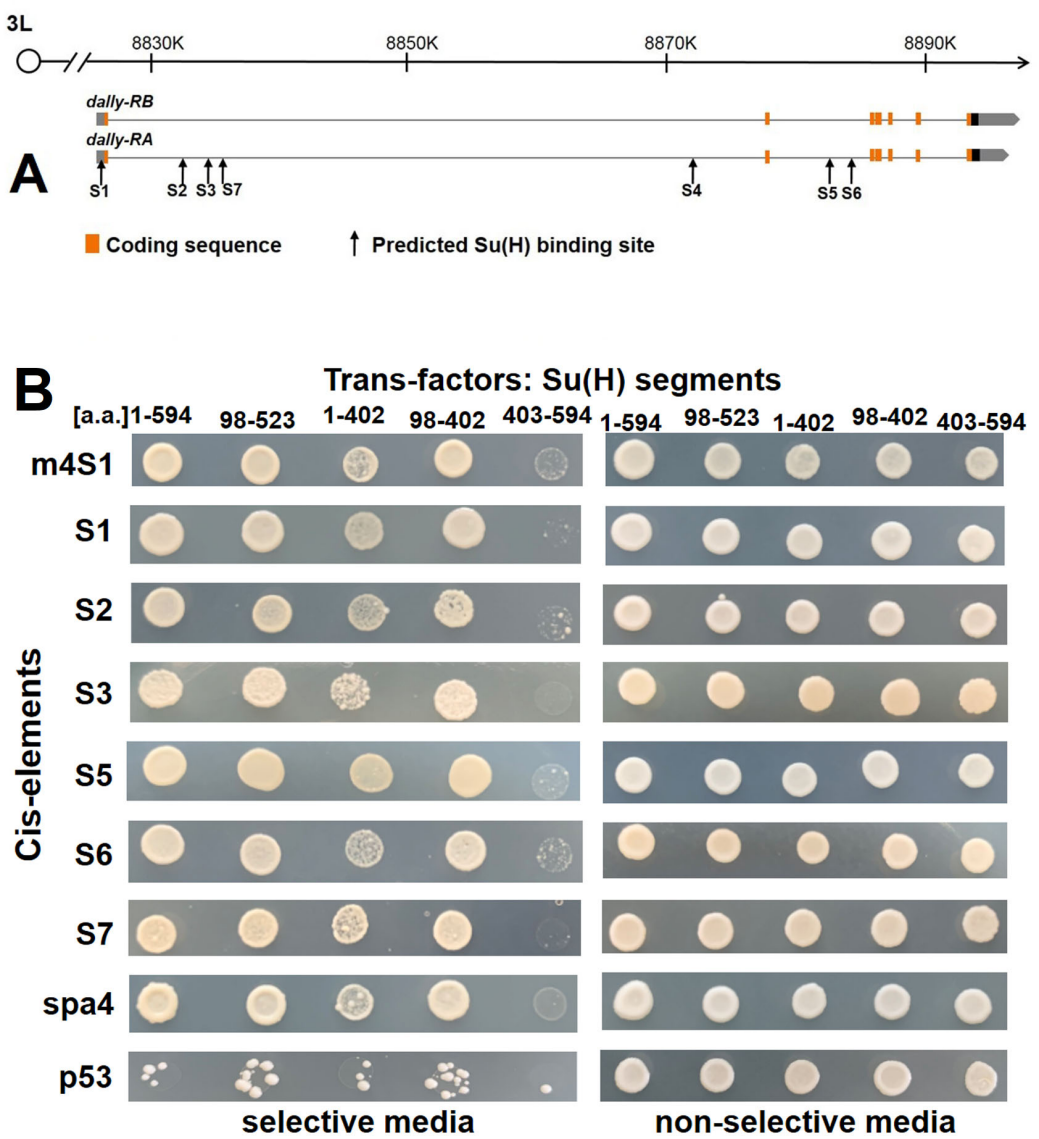
**Y1H assay of the putative Su(H) binding sites in *dally* genomic region.** (A) The positions of the putative Su(H) binding sites S1–7 in the genomic region of *dally.* (B) S1–3 and 5–7: six of the seven putative Su(H) binding sites from the *dally* genomic region were qualified to be tested in Y1H assay, in which protein-DNA binding activity is reflected by colony growth. m4S1 from *E(spl)-C* and spa4 from *dPax2* served as the positive controls for binding with Su(H), whereas sequences from *p53* served as the negative control. Different constructs of Su(H) protein were used to show whether or not the DNA-binding activity was associated with the presence of the DNA-binding domain of Su(H). The segment of 403-594 a.a. is the only one devoid of DNA-binding domain. The cell growth on non-selective media validates that the presence of these cis- and trans-factors did not cause poor growth on their own.

To investigate if Su(H) regulates Dally expression directly, we scanned the genomic region of *dally* for the putative binding sites of Su(H). Based on the reported consensus sequences of Su(H) (Rebeiz et al., 2002), we identified seven putative binding sites which we designated as S1–7 (Fig. 5A, see Materials and Methods for sequence details). All of them are located in the non-coding region either in 5′UTR (S1) or in the introns (S2–7) without clustering. We employed a Yeast-1-hybrid (Y1H) assay to detect whether there is a direct interaction between Su(H) protein and these predicted sites (Fig. 5B). In Y1H assay, protein-DNA binding activity is reflected by the colony growth. The functional domain structure of Su(H) has been well characterized previously (Kovall and Hendrickson, 2004; Yuan et al., 2016). All segments of Su(H) that contained DNA-binding domain could interact with all putative sites tested; whereas the C-terminal segment (403-594a.a.) without DNA-binding domain showed very poor binding (reflected by poor colony growth) to these sites. This assay implies that Su(H) can directly interact with these putative sites in a manner dependent on the DNA-binding domain.

### Functional assay of human GPCs and disease-associated mutant forms

GPC1-6 are the six members of glypican family in human genome, and grouped into two subfamilies: GPC1/2/4/6 and GPC3/5 (Filmus et al., 2008). The primary structures of glypicans are conserved from fruit fly to human with ∼40% similarity between Dally and GPC3 or 5 (Fig. S2). However, the function of glypican is elaborated and complicated by various large sugary modifications of the protein core, and how glypican structure relates to the function in different cellular contexts or how the mutant forms lead to anomalies is difficult to clarify.

**Fig. 6.**
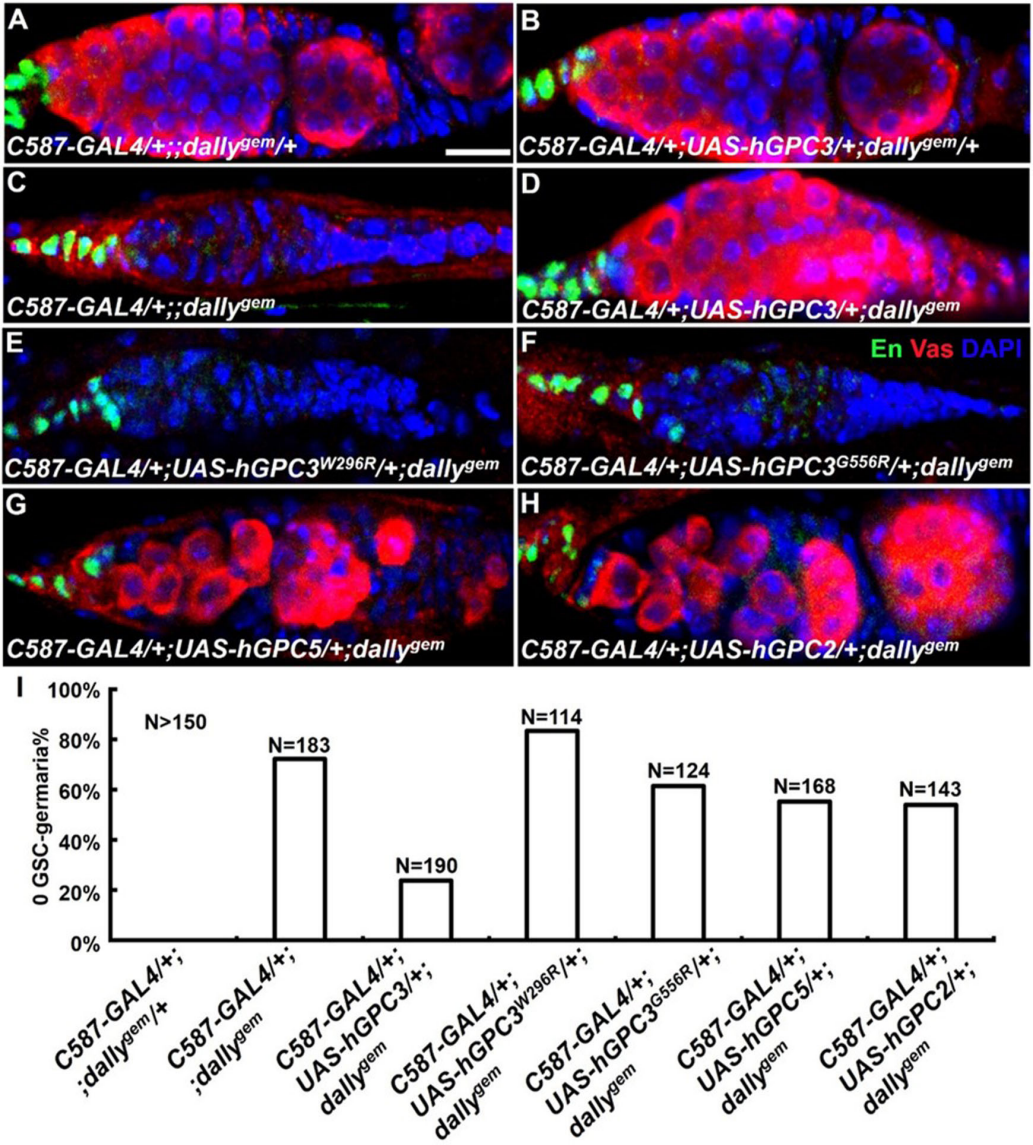
**Human glypicans restored the germline stem cells in *dally* mutant ovaries.** Color code for immunostained signals is shown in panel F. (A,B) The control germarium is filled with Vas-positive germ cells. (C) The *dally^gem^* germarium lost all germ cells, including GSC. (D) Germline loss was rescued by human *GPC3*. (E,F) Germline loss was not rescued by the mutant forms of human *GPC3*. (G) Germline loss was partially rescued by human *GPC5*. (H) Germline loss was partially rescued by human *GPC2*. (I) Percentage of the 0 GSC-germaria shown in panel A-H. N, number of gemaria scored. Empty germarium without Vas-positive single cells was counted as ‘0 GSC’. Scale bar: 10 μm.

To test if the ovarian GSC niche is an effective readout to reveal the structure–function relationship of human glypicans, we expressed human GPC2, 3 or 5 in fly ovary to see whether it can substitute the function of its fly homolog (Fig. 6). Although GPC3 and GPC5 are similarly close to Dally at the level of amino acid sequences, GPC3's rescuing activity for *dally* mutant was higher than GPC5 (Fig. 6I). As expected, GPC3's activity was diminished by a point mutation (W296R) that alters an amino acid conserved from worm to human and is also associated with SGBS overgrowth (Veugelers et al., 2000); or by another residue change (G556R) which disrupted the protein's anchoring to the cell surface and is associated with artery anomaly (Penisson-Besnier et al., 2008; Shi and Filmus, 2009) (Fig. S2; Fig. 6E,F,I). Sharing 30% similarity with Dally and belonging to a different subgroup, GPC2 showed lower activity in the GSC niche than GPC3, similar to GPC5 (Fig. 6I). Taken together, the ovarian GSC niche can serve as an effective readout to dissect the structure–function relationship of human glypicans.

## DISCUSSION

We have provided genetic evidence to illustrate that the Notch pathway specifies the range of a stem cell niche via controlling the expression pattern of the cell surface glypican (schematic model shown in Fig. S3). Additionally, we have validated the germline stem cell niche in *Drosophila* ovary as an informative readout for analyzing human glypicans. The current data lead to more questions to be resolved.

### How is Dally expression restricted to the GSC niche?

EGFR signaling has been found to repress Dally expression in germarium posterior of GSC niche (Liu et al., 2010b), without learning how Dally expression is activated. As shown in Fig. S1, Notch expression is present ubiquitously in pre-pupal ovary when the GSC niche is forming. By immunostaining, the activated form of Notch (the intracellular domain released from cell membrane and translocated to the nucleus) was not detectably higher in the nuclei of cap cells (Fig. S1E), whereas Dl (a Notch ligand) was only expressed in the terminal filament (Fig. S1A). Since Dl is a cell-membrane-bound ligand, it only activates Notch on the cells in contact. If the Dl–Notch pair is sufficient to activate Dally expression, we expect to detect Dally in the terminal filament and in one surrounding layer of cells, including cap cells adjacent to the posterior end of the terminal filament. However, we only observed Dally transcription in the cap cells (Guo and Wang, 2009). Consistently, one of the Notch targets is also transcriptionally activated only in cap cells (Shimizu et al., 2017). This could be explained by either additional positive regulator(s) required in the cap cells or negative one(s) present in the terminal filament to restrict Dally expression specifically to the cap cells. Notably, a large Maf transcription factor, Tj, is required for cap cell fate by blocking TF formation (Panchal et al., 2017). Recently, a steroid-*miR-125*-Tom-Neur-Delta-Notch signaling cascade has been reported to be responsible for the restricted activation of the Notch pathway in the posterior part of the terminal filament (Yatsenko and Shcherbata, 2018). However, this model cannot explain how Dally is only expressed in cap cells. Thus, our speculation needs further investigation.

### Functional activity of human glypicans assayed in a heterologous system

What constitutes the functional activity of different glypicans serving in a developmental or cellular context? How do we interpret the results of human glypicans obtained in fruit fly? Since glypicans can interact with multiple signaling pathways not necessarily through the same set of structural features, we restrict our discussion in relation to BMP, the signal molecule most relevant to the ovarian GSC niche (Guo and Wang, 2009; Hayashi et al., 2009). In this biological context, human GPC3 substituted its *Drosophila* counterpart with an activity stronger than GPC5 or GPC2 (Fig. 6), though the primary structures of GPC3 and GPC5 are similarly close to Dally. While the core protein folding of glypicans is alike due to the 14 conserved cysteine residues (Filmus et al., 2008), this suggests that the differential activities between GPC3 and GPC5 may reflect the different modifications by the heparan-sulfate chains. Indeed, in an *in vitro* system mimicking the ovarian GSC niche, heparan sulfate is required for Dally to mediate trans-signaling of BMP in adjacent cells (Dejima et al., 2011). GPC3 and GPC5 have been shown to carry different numbers of sugar chains or different sites of sulfation (N- versus non-N-sulfated) (Li et al., 2011; Filmus and Capurro, 2014), whether these differences contribute to glypican's activity in BMP trans-signaling remains to be investigated.

Emerging literature has discussed the possibility of glypicans as both diagnostic markers and therapeutic targets for cancer (Filmus and Capurro, 2013; Bosse et al., 2017; Fu et al., 2018; Li et al., 2018). Is it reasonable to employ this *Drosophila* structure in the screen of therapeutic agents? Theoretically, it could be a valid screen if BMP signaling and glypican levels are altered in tumorigenesis. Although it is clear in the *Drosophila* ovarian niche that glypican is pro-BMP signaling, glypican's role in developmental growth or tumorigenesis in different organs seems much more complicated or even contradictory. The evidence that glypicans interact with signaling molecules is consistent in different experimental systems by different research groups. The biological consequence really depends on the spatial relationship between glypican and the source of a signal, or whether glypican is anchored or released from cell surface. Thus, it is critical to clarify these issues before an effective screen can be designed.

## MATERIALS AND METHODS

### Fly strains

*P[PZ]dally^06464^*, *bab1-GAL4* (BL#6803), *UAS-Notch RNAi* (BL#7078) and balancers were obtained from Bloomington *Drosophila* Stock Center (BDSC); *UAS-Su(H)-RNAi* (NIG#3497R-1) from NIG-Fly. *C587-GAL4, UAS-Dl* from Ting Xie (Kawase et al., 2004); *dally^gem^* from Hiroshi Nakato (Nakato et al., 1995; Takeo et al., 2005); *UAS-dally* (Guo and Wang, 2009). None of the *dally* alleles used in this study is a strict null or amorph.

### Transgenic flies

The cDNA of *hGPC3* (Genebank: NM_004484.3), *hGPC5* (Genebank: NM_004466.6) and *hGPC2* (Genebank: NM_152742.2) were cloned from HEK 293 cells or HeLa cells, and cloned into UAS vector. *UAS-hGPC3^G556R^* was generated by inverse PCR-based site-directed mutagenesis (TOYOBO, SMK-101). The *w^1118^* and *p51D* stocks were chosen as the hosts for P-element and attB**-**attP mediated transgenesis, respectively (Rubin and Spradling, 1982; Bischof et al., 2007).

### Immunohistochemistry and microscopy

All samples were dissected in PBS, fixed and stained as described previously (Li et al., 2007). Primary antibodies were used at the following dilutions: mouse anti-En [1:50, Developmental Studies of Hybridoma Bank (DSHB)]; rabbit anti-Vasa (1:4000, against peptide MSDDWDDEPIVDTRGARC); mouse anti-β-gal (1:100, DSHB, 40-1a); rabbit anti-β-Gal (1:50000, Cappel); Delta (1:200, DSHB, C594.9B); NECD (1:50, DSHB, 458.2H); NICD (1:50, DSHB, C17.9C6); LaminC (1:200, DSHB). Alexa-Fluor-conjugated secondary antibodies were used at 1:4000 (Molecular Probes, Invitrogen). Fluorescent images were collected by OLYMPUS FV1000 Confocal microimaging system.

### Y1H

To detect putative DNA-binding sites of Su(H) in the genomic region of *dally*, we screened six cis-elements containing the consensus sequence for Su(H) using Clonetech kit (Matchmaker^®^ Gold Yeast One-Hybrid Library Screening System, cat#630491). The three different segments of Su(H) tested were: 1-402aa, 98-402aa and 403-594aa. The cis-element sequences cloned into the reporter vector were: m4S1 [a known Su(H) target site from *E(spl)-C*], caccgagt gtgggaaa ctac; spa4 [a known Su(H) target site from *dPax2*] (Flores et al., 2000), aaata tatgggaa cacagat; *dally*-S1, atactt gtgtgaaa tttagc; *dally*-S2, tcagtc gttcccac acgcag; *dally*-S3, ctaagac gtgggaaa agcac; *dally*-S5, ccaaggc gtgggaaa cagca; *dally*-S6, tgtgtgt gtgagaaa tcaca; *dally*-S7, atcgat ttcacacg catata. S4 could not be tested in this Y1H system due to very high constitutive activation without adding any trans-factors. p53 cis-element was provided by the Clonetech kit.

## Supplementary Material

Supplementary information
