# Supplementary material for: Notch signaling governs the expression of glypican Dally to define the stem cell niche
Source: Biol Open. 2020 Jan 10;9(1):bio047696. doi: 10.1242/bio.047696 (PMC6994927; doi:10.1242/bio.047696)
Supplement: Supplementary information [file biolopen-9-047696-s1.pdf]

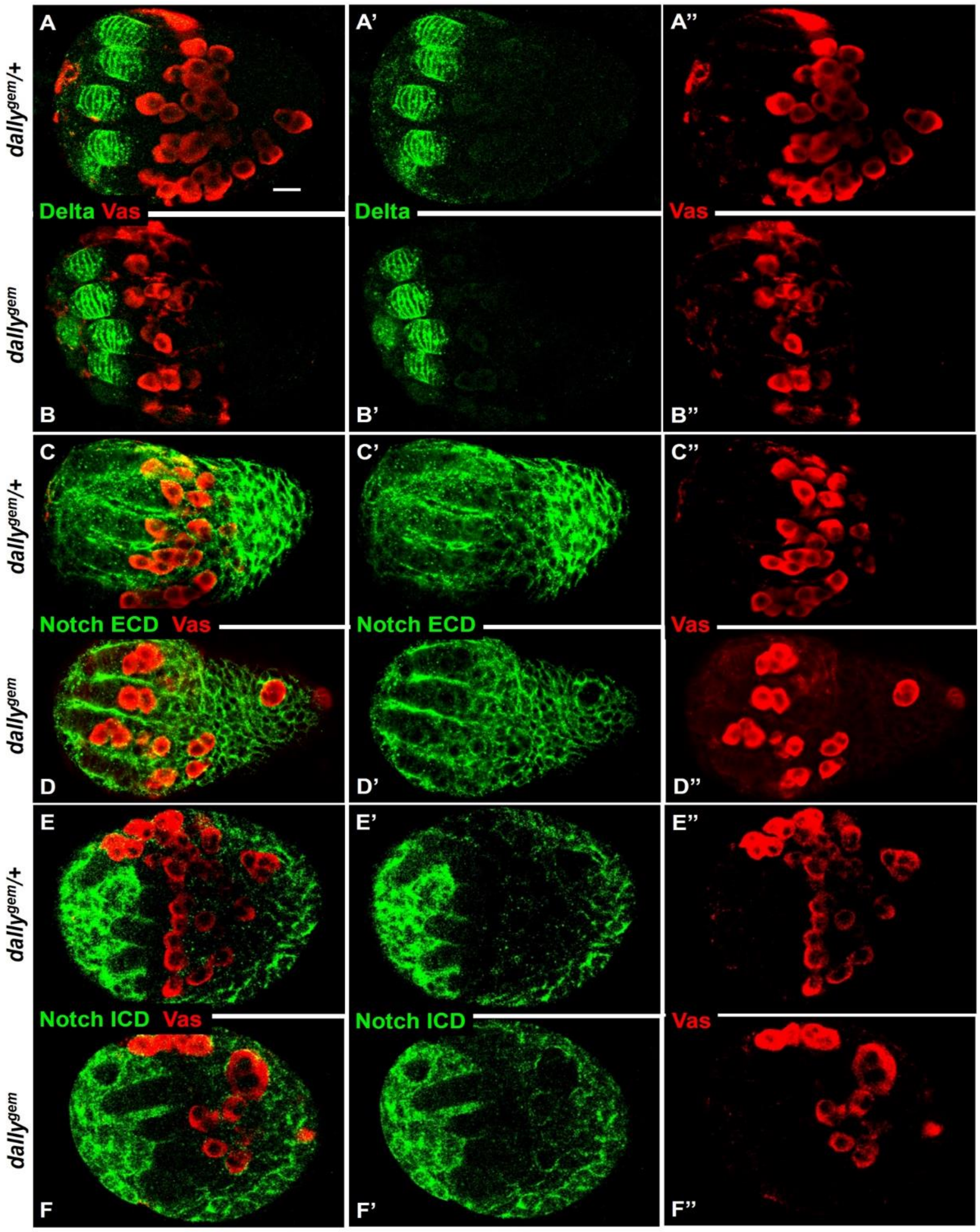

Fig S1. Immunestaining of Notch pathway components showed no obvious change in ovarian GSC niche of *dally* mutant.

Early pupal ovaries are shown. (A, C and E) Genotype: *dally*^{gem}/+. (B, D and F) Vas-positive germ cells decreased in *dally* mutant (*dally*^{gem}) ovary. (A and B) Delta, one of the Notch pathway ligands, is expressed in the terminal filament. (C and D) The pattern of Notch ECD (antibody against EGF-like repeats #12-20 in the extracellular domain of Notch). (E and F) The pattern of Notch ICD (antibody against Notch intracellular domain, a.a. 1791-2504). Bar, 10μm.

Biology Open • Supplementary information

```
hGPC2   ------MSALRPLLLLLLPLCPGPGPGPGS------------------------------ 24
DALLY   --MAARSVRLAQLLLFTL-LCGFVGLSAAKHLDLDGIHHHQHHLHSATTHHRRRLQRDSR 57
hGPC3   ---MAGTVRTACLVVAMLLSLDFPGQA--------------------------------- 24
hGPC5   MDAQTWPVGFRCLLLL-----ALVGSA--------------------------------- 22
                    *::         * .

hGPC2   ----EAKVTRSCAETRQVLGARG-YSLNLIPPALISGEHLRVCP-QEYTCCSSETEQRLI 78
DALLY   AKDAVGGSTHQCDAVKSYFESIDI---------KSSGTYSEKGAICGGNCCNNATELELR 108
hGPC3   -QPPPPPPDATCHQVRSFFQ-RLQPGLKWVPETPVPGSDLQVCLPKGPTCCSRKMEEKYQ 82
hGPC5   ----RSEGVQTCEEVRKLFQWRLLGAVRGLPDSPRAGPDLQVCISKKPTCCTRKMEERYQ 78
                   *  .:. :                 *   .       .**.   * .

hGPC2   RETEATFRGLVEDSGSFLVHTLAARHRKFDEFFLEMLSVAQHSLTQLFSHSYGRLYAQHA 138
DALLY   DKAAGMFEQLLHHHTSSLRGVLETNAKQFQSHVLELAQISENMTHSLFSKVYTRMVPSSR 168
hGPC3   LTARLNMEQLLQSASMELKFLIIQNAAVFQEAFEIVVRHAKNYTNAMFKNNYPSLTPQAF 142
hGPC5   IAARQDMQQFLQTSSSTLKFLISRNAAAFQETLETLIKQAENYTSILFCSTYRNMALEAA 138
          :   :. ::.     *   :  .   *:. .  :   :::    :*   *  :  .

hGPC2   LIFNGLFSRLRDFYG-------------ESG-EGLDDTLADFWAQLLERVFPLLHPQYSF 184
DALLY   MMIHQLYTEIMNHLIYTSNYTNSNGQLGRRGIGSVQSNLEEAVRHFFVQLFPVAYHQMVH 228
hGPC3   EFVGEFFTDVSLYIL------------------GSDINVDDMVNELFDSLFPVIYTQLMN 184
hGPC5   ASVQEFFTDVGLYLF------------------GADVNPEEFVNRFFDSLFPLVYNHLIN 180
          .  ::: :  .                    . : .  :   .::  :**: : :

hGPC2   ---------PPDYLLCLSRLASSTDGSLQPFGDSPRRLRLQITRTLVAARAFVQGLETGR 235
DALLY   LSKNNLGDLHEDYVNCLQ----HNFDEMHPFGDIPQQVQSNLGKSVHMSNVFMNALLQAA 284
hGPC3   ---PGLPDSALDINECLR----GARRDLKVFGNFPKLIMTQVSKSLQVTRIFLQALNLGI 237
hGPC5   ---PGVTDSSLEYSECIR----MARRDVSPFGNIPQRVMGQMGRSLLPSRTFLQALNLGI 233
                   :   *:         .:  **: *: :  :: :::  :. *::.*  .

hGPC2   NVVSEALKV---PVSEGCSQALMRLIGCPLCRGVP-----SLMPCQGFCLNVVRGCLSSR 287
DALLY   EVLSEADALYGEQLTDTCKLHLLKMHYCPNCNGHHSSSRSETKLCYGYCKNVMRGCSAEY 344
hGPC3   EVINTTDHL---KFSKDCGRMLTRMWYCSYCQGLM-----MVKPCGGYCNVVMQGCMAGV 289
hGPC5   EVINTTDYL---HFSKECSRALLKMQYCPHCQGLA-----LTKPCMGYCLNVMRGCLAHM 285
        :*:. :  :    .:. *   * ::  *  *.*           * *:*  *::** :

hGPC2   G--LEPDWGNYLDGLLILADKL-QGPFSFELTAESIGVKISEGLMYLQENSAKVSAQVFQ 344
DALLY   AGLLDSPWSGVVDSLNNLVTTHILSDTGIINVIKHLQTYFSEAIMAAMHNGPELEKKVKK 404
hGPC3   VE-IDKYWREYILSLEELVNGM-YRIYDMENVLLGLFSTIHDSIQYVQKNAGKLTTTIGK 347
hGPC5   AE-LNPHWHAYIRSLEELSDAM-HGTYDIGHVLLNFHLLVNDAVLQAHLNGQKLLEQVNR 343
           ::  *   :  .*  *         .:  .   :   . :.:     *. ::   :  :

hGPC2   ECGPPDPVPARNRRA--PPPREEAGRLWSMVTEEERPTTAAGTNLHRLVWELRERLARMR 402
DALLY   TCGTPSLTPYSSGEPDARPPPHKNNVKWATDP-------------DPGMVLFLSTIDKSK 451
hGPC3   LCAHSQQRQYRSAYYPEDLFIDKKVLKVAHVEHEETL--------SSRRRELIQKLKSFI 399
hGPC5   ICGRPVRTPTQSPRCSFDQSKEKHGMKTTTRNSEETL--------ANRRKEFINSLRLYR 395
         *.        .         .:     :                      : . :

hGPC2   GFWARLSLTVCGDSRMAADASLEAAPCWTGAGR-GRYLPPVVGGSPAEQVNNPELKVDAS 461
DALLY   EFYTTIVDNFCDEQQHSR----DDHSCWSGDRF-GDYTQLLINPGTDSQRYNPEVPFNAK 506
hGPC3   SFYSALPGYICSHSPVAE----NDTLCWNGQELVERYSQKAARNGMKNQFNLHELKMKG- 454
hGPC5   SFYGGLADQLCANELAAA----DGLPCWNGEDIVKSYTQRVVGNGIKAQSGNPEVKVKG- 450
         *:  :   .* ..  :     :   **.*      *       .   *    *: ...

hGPC2   GPDVPTRRRRLQLRAATARMKTAALGHD------LD-----------------GQDADED 498
DALLY   AQTGKLNELVDKLFKIRKSIGAAAPSNSIQATHDIQNDMGEGSGG---GEGQIGDDEEEY 563
hGPC3   -PEPVVSQIIDKLKHINQLLRTMSMPKG----RVLDKNLDE----EGFESGDCGDDEDEC 505
hGPC5   -IDPVINQIIDKLKHVVQLLQGRSPKPD----KWELLQLGSGGGMVEQVSGDCDDE-DG- 503
               .   :*      :   :   .                         .:: :

hGPC2   ASGSGGG------QQYADDWMAGAVAPPARPPRPPYPPRRDGSGGKGGGGSARYNQGRSR 552
DALLY   GGAHGSGDGSGDGPHTPI---------EESEGTTTNEVESRDS------G-----KT--- 600
hGPC3   IGGSGDGMIKVKNQLRFLAELAYDLDVDDAPGNSQQATPKDNE------ISTFHNLG--- 556
hGPC5   CGGSGSGEVKR--TLKITDWMPDDMNFSDVKQIHQTDT-----------GSTLDTTG--- 547
         .. *.*

hGPC2   SGGASIGFHTQTILILSLSALALLGPR--            579
DALLY   SGSNPL-EGTATWMLLTLVTMLFSSCS--            626
hGPC3   NVHSPL-KLL-TSMAISVVCFFFLVH---            580
hGPC5   AGCA---VAT-ESMTFTLISVVMLLPGIW            572
                     : :::  . :
```

W296R
G556R
(Simpson-Golabi-Behmel Syndrome)

Fig S2. Predicted amino acid sequence of Dally aligned with human GPCs. “*” indicates identical residues; “:”, at least 50% identical; “.”, at least 50% similar. Rectangles mark the residues associated with SGBS and tested in this report.

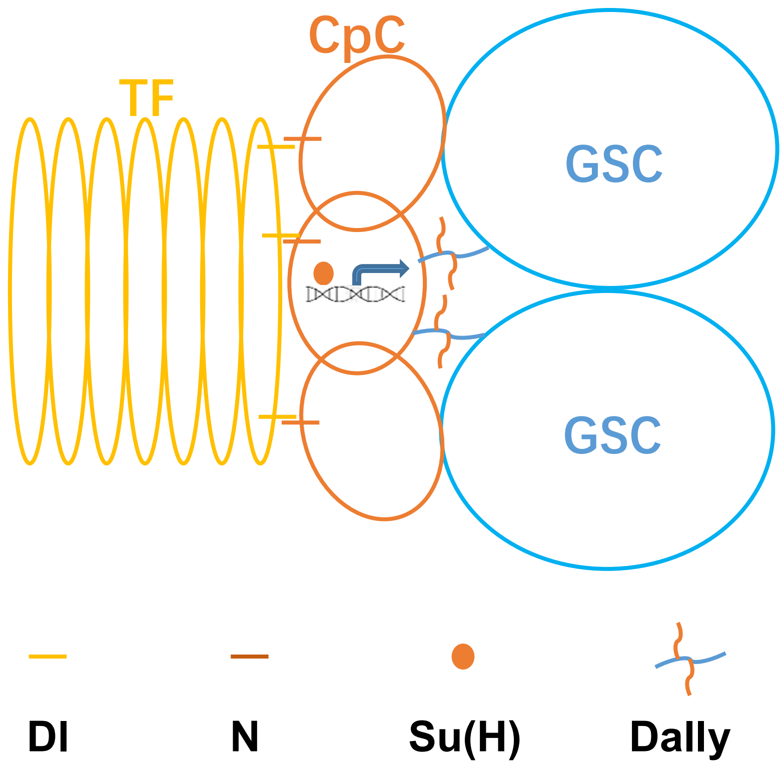

Fig S3. Schematic model of Notch signaling and Dally expression in the ovarian stem cell niche.

TF, terminal filament; CpC, cap cell; GSC, germline stem cell.

Biology Open • Supplementary information
